# ELCID: early lung cancer identification and diagnosis - an embedded interview study to explore patient participation and recruitment

**DOI:** 10.1186/1745-6215-14-S1-P102

**Published:** 2013-11-29

**Authors:** Hayley Prout, Richard Neal, Kirsty Roberts, Chris Hurt, Trevor Rogers, Willie Hamilton, Rhiannon Tudor Edwards, Angela Todd, David Parker, Emma Thomas Jones, Kerenza Hood, Gareth Griffiths, Allan Barham, Jim Fitzgibbon, Annmarie Nelson

**Affiliations:** 1 Marie Curie Palliative Care Research Centre, Wales Cancer Trials Unit, Cardiff University, Cardiff, UK; 2 North Wales Centre for Primary Care Research, College of Health & Behavioural Sciences, Bangor University, Wrexham, UK; 3 Wales Cancer Trials Unit, Cardiff University, Cardiff, UK; 4 Doncaster Royal Infirmary, Doncaster, UK; 5 University of Exeter Medical School, Exeter, UK; 6 Centre for Economics and Policy in Health, Bangor University, Bangor, UK; 7 Centre for Health and Social Care Research, Sheffield Hallam University, Sheffield, UK; 8 Wrexham Maelor Hospital, Wrexham, UK; 9 South East Wales Trials Unit, Cardiff University, Cardiff, UK

## Background

The ELCID Trial is a feasibility randomised controlled trial examining the effect on lung cancer diagnosis of giving an urgent chest x-ray to smokers, and recent ex-smokers, aged over 60 with new chest symptoms.

## Aims

The qualitative component explores the feasibility of individually randomising patients to an urgent CXR or not and investigates any barriers to patient participation.

## Methods

To date we have conducted semi-structured interviews with six primary care staff (practice managers, research nurses), ten patients randomised to ‘extra-NICE' guidelines for referral for urgent chest x-ray, and six patients randomised to ‘usual care' (NICE guidelines)). We hope to also interview patients who decline randomisation. Interviews were analysed using a Framework approach.

## Results

Initial analysis indicated that practices have struggled to recruit patients, partly due to the eligibility criteria that requires ex-smokers to have stopped smoking within the last five years. Practices with a research nurse have recruited the most patients. Patients indicated that they are happy to take part in the trial and their anxiety levels were not raised. Most patients hoped to be randomised to urgent chest x-ray, although those who were not did not go back to their GP to request one.

## Conclusions

Eligibility criteria needed revision to include ex-smokers of any duration. These preliminary findings suggest that the trial appears to be feasible and patients are happy to accept randomisation. The findings will inform the design of the main trial in the future.

